# Letter from A. Lagorio

**Published:** 1887-01

**Authors:** A. Lagorio

**Affiliations:** Chiavari, Italy


### FOREIGN CORRESPONDENCE.

*To the Editors of the* CHICAGO MEDICAL JOURNAL AND EXAMINER.

*Gentlemen:*

Professor Mariano Semmola, of Naples, is a man whom Italy delights to own and to honor. He is no stranger to the medical world, his works are renowned for thoroughness in investigation and originality. Anything from his pen is worthy of special attention. In 1883, the learned professor read before the Academy of Medicine, of Paris, an elaborate and scientific paper on the hæmatic and discrasic origin of Bright's disease. Although at that time his address received great attention, it yet met with some opposition. But this did not deter him from adhering to his theory. To convince his opponents he immediately began another series of experiments which impressed him more fully with the truth of his views. He then sent another communication to the academy on the same subject, which, being of special interest to the students of renal pathology, I have endeavored to summarize.

Professor Semmola believes that the elimination of albumen from the organism is due to its pathological diffusibility. Albuminuria happens without any previous alteration of the epithelium of the tubes, or of the glomeruli of the kidneys. It is nothing but a natural and a necessary effort of the organism to expel what is foreign to itself. But if this eliminating functional work continues, the kidneys become the seat of a morbid change, from a simple hyperæmia to a diffused nephritis. Professor Semmola was enabled to produce a true experimental nephritis with injections of albumen taken from patients having Bright's disease. The contradictory results so far obtained with the injections of blood serum are easily explained, for the fundamental condition, so far as known, which causes the elimination of the albuminoids of the blood, resides in their pathological diffusibility. Before making any injection it is necessary to know the proportion of the dialysable albuminoids contained in it, and with this simple knowledge we can easily foretell if there will be any albuminuria after the injections. This is the starting point of any conclusion as to the assimilability or not of the albuminoids. In infectious diseases, as smallpox, scarlet fever and diphtheria, there has been found in the blood during the first few days a large quantity of dialysable albuminoids (15 to 22 per centum), and we cannot believe that this albuminuria is due to any nephritis. The old authors were in the right, believing it of a discrasic origin.

The subcutaneous injections of egg albumen, if continued five or six days, will produce a true albuminous dyscrasia, so that the total quantity of albumen eliminated day by day through the different channels becomes larger than the amount which was injected. Gradually, as the albuminous dyscrasia is produced, and the elimination by the urinary tract increases, there is also a decrease in the quantity of urea eliminated, notwithstanding that the regimen and other conditions of life of the animal have not been changed. After fifteen or twenty days of these albuminous injections we can easily detect, besides the albuminous dyscrasia, a serous infiltration (anasarca) and light dropsies of the cavities. Do not such effects show the experimental production of a genuine form of Bright's disease? 1st, albuminuria; 2d, albuminous dyscrasia, with progressive diffusibility of the albuminoids of the blood; 3d, diminution of urea production; 4th, dropsy; 5th, nephritis.

The only important factor which is defective in this succession of phenomena in the dogs submitted to experiment is the progressive enfeeblement of their cutaneous function, coming on in consequence of the action of humid cold. But that which in similar circumstances experimental pathology cannot give us we get from clinical experience, when, for example, we find in a scarlatinous patient symptoms of albuminuria during the stage of desquamation preceding or accompanying convalescence. This albuminuria, which we might call post-scarlatinous, is of much greater gravity than that which comes on in the acute stage of scarlatina. This same clinical experience gives us valuable ideas on the pathogeny of Bright's disease, be it acute or chronic. The acute is always due to a rapid chilling of the skin, and in consequence the congestive effects on the kidneys are considerable, and prevail on the dyscrasic effects which at this stage are not manifest. On the contrary, in the chronic the effects of humid cold being excessively slow, the mechanical effects on the kidneys will be insignificant, while the dyscrasic effects are greater. In the acute they are rapid, and therefore their evolution and curability exceptionally leave the limit of curability of the ordinary acute diseases; but in the chronic they develop slowly and without any knowledge on the part of the patient. Latent and insidious, little by little, they affect the principal seats of nutrition, that is, the chemico-biological constitution of the albuminoids, producing chronic Bright's disease. From all his researches Professor Semmola has drawn these conclusions: 1st. For the etiology, the excessively slow action of humid cold on the skin. 2d. For the alterations, a progressive diminution of the cutaneous function, due to atrophy of the sudoriferous glands, or to changes of the malpighian layer. 3d. A chemico-molecular alteration of the albuminoids due to the alimentation, an alteration characterized by their pathological diffusibility and, in consequence, by their non-assimilability, and by their necessary diminution through all the emunctories, principally the kidneys. 4th. A progressive diminution of combustion of the albuminoids, which is indicated by a progressive lessening in the formation and elimination of urea. 5th. A serous, subcutaneous infiltration, commencing in the face with erratic and progressive characters, but excessively slow, not in keeping with the hydroæmia. 6th. A characteristic cachexia not in relation with the loss of albumen, but with a general defect of assimilation. 7th. The secondary development of an inflammatory process of both kidneys at the same time, an excessively slow process affecting the organs with the histological characters of a diffused nephritis.

Regarding the *unity* of the disease, Professor Semmola is a strong opponent of those who formulate their theory with the words: *clinical unity* and *anatomical plurality*. He says: "It is impossible to admit the principle of the clinical unity with the plurality of the anatomical lesions. The empirical acceptation of the name of Bright's disease applied to completely different diseases would not honor the discovery of the celebrated English physician; the true chronic disease of Bright is a constant, well-marked, morbid type, and agrees with the etiology, evolution, pathology and treatment."

Now as to treatment: Since 1861 Professor Semmola, believing in the secondary character of the renal process and in the great importance of the cutaneous functions, pathogenically as well as curatively, obtained some complete cures with the hydro-sudoriferous plan of treatment. To-day the majority of clinicians recognize the possibility of curing the disease when it has not yet reached the confirmed anatomical stage, that is, when there has not occurred in the kidneys any destruction of some histological elements indispensable to their function. The following are the fundamental indications for treatment:

1st. To nourish the patients with easily digested substances.

2d. To methodically assist the cutaneous functions by stimulating the vitality of the skin.

3d. To favor by every possible means the assimilation, combustion and elimination of the albuminoids introduced into the organism.

The treatment of Bright's disease during its long period of curability ought to be: 1st. Exclusive lacteal regimen. Milk acts in a wonderful manner in these patients, not only because of its diuretic properties, but still more because of its assimilability. It ought to be continued for a long time. 2d. Methodical and repeated impressions on the skin by dry friction, by massage, and frequently by means of hot-air sweatings. Hydrotherapy is not well tolerated by patients because of the little functional and sanguineous reaction of the skin. 3d. Patients must live in temperate and dry surroundings. This is the *sine qua non* condition of success. In winter, and especially in the variable climates, the patient must substitute room gymnastics for walks in the open air 4th. Administer the iodide and the chloride of sodium in progressive doses according to tolerance. 5th. If after two or three weeks the albumen has not yet completely disappeared, although there be no traces of anasarca, the iodide of sodium ought to be exchanged for the phosphate of soda or of lime (3 to 4 grains in 24 hours). 6th. Methodical inhalations of oxygen; the albumen sometimes disappears in a few days, while the morphological elements persist for some time in the urine. 7th. Do not use astringents (gallic acid, perchloride of iron, acetate of lead), they are of no benefit, but harmful; for, admitting that these remedies have an astringent action in all sanguineous and capillary network, the capillaries of the skin must also feel the effect, and such an action will only produce a considerable enfeeblement of the cutaneous functions. These astringents would act in a manner precisely opposite to that which forms the principal plan of treatment of Bright's disease, according to the opinion of all physicians without distinction of doctrine.

Those who find in anatomical lesions of the kidneys the only cause of Bright's disease, will do well to ponder over Professor Semmola's theory.

I have just received the mournful news of the death of Professor Felice Margary of Turin. He died of intestinal cancer. He was among the most eminent surgeons of Italy, certainly the best on orthopœdic surgery. In 1863 he entered the major hospital as an assistant physician; in 1868 was nominated assistant surgeon; in 1879 was promoted chief surgeon, which important office he held till his death. He possessed a wide European reputation for his skillful orthopœdic operations, obtaining wonderful results. For the past three years he was an associate editor of the *Archivio di Ortopedia.* There appeared in the 2d, 3d and 4th numbers his last and most important article, giving the history and successful results which he had obtained in 207 osteotomies for the relief of genu-valgum, 83 for genu-varum and rachitic curvatures of the bones of the inferior limbs, 18 for angular position of the knee, 12 for deformities of the hip, 23 for equinus feet, equino-vari, valgi, etc., etc.

The poor will most keenly feel his loss. After operating on them, he would support them financially and otherwise through their illness, and tender in every way paternal attention and help. He was the admiration of his assistants and students, for few teachers were equal to him in brilliancy of oratory and learning. The funeral ceremonies were solemn and impressive, worthy in every way of the great surgeon.

A. LAGORIO.

Chiavari, Italy, December 2nd, 1886.

